# Maternal Love in Nature

**Published:** 1861-01

**Authors:** J. L. C. Schroeder van der Kolk, William Daniel Moore

**Affiliations:** Professor in the University of Utrecht.; Honorary Member of the Swedish Society of Physicians, and of the Norwegian Medical Society.


### Art. IX.—MATERNAL LOVE IN NATURE;

By J. L. C. Schroeder van der Kolk, Professor in the University of Utrecht.

Translated from the Original Dutch by William Daniel Moore, M.D., M.R.I.A., Honorary Member of the Swedish Society of Physicians, and of the Norwegian Medical Society.

### TRANSLATOR'S PREFACE.

In the course of a lengthened correspondence which it has been my privilege to enjoy with the eminent Utrecht Professor, Heer J. L. C. Schroeder van der Kolk, in reference to an important physiological work of his, which I have translated and edited for the New Sydenham Society, the author was good enough to send me, with some other minor essays, a copy of a dissertation read by him before the Physical Society of Utrecht, at one of the meetings to which ladies are admitted, on "*Maternal Love in Nature*."

It appears to me that the distinguished author has, in this essay, with peculiar force and beauty, shown, that in the works of nature the great truth that "God is love," is exhibited in a manner second only to its demonstration in the all-important fact, declared by the Saviour himself, that "God so loved the world, that he gave his only begotten Son, that whosoever believeth in him should not perish, but have everlasting life." I have therefore thought that an English version of the essay might be not unacceptable to my fellow-countrymen.

The subject specially chosen by the author for the illustration of the statement just quoted, is the means taken by the parents, in every class in the animal kingdom, for the preservation of their young. This is beautifully traced in the original essay (and I trust I have succeeded in the following pages in faithfully conveying the writer's meaning, however much his clear and energetic style may have suffered in the translation), from the insect which providently lays up for the offspring it is never to behold, a sufficient store of the necessary food, to the Christian mother, who provides not merely for the temporal wants of her children, but endeavours also to feed them with "the meat which endureth unto everlasting life," by unfolding to them, so far as that glorious theme can be comprehended here below, the vastness of redeeming love, and by seeking to excite in their youthful breasts that response of faith, and hope, and love, which alone can form the basis of all true morality.

---

When we attentively survey the economy of nature, and contemplate the profusion of life everywhere scattered under the most conflicting forces and influences; the unity of object combined with the greatest diversity of means, and the most steadfast order observed amidst the greatest apparent confusion, constrain us to admiration and reflection. We see among the myriads of creatures inhabiting the surface of the earth, an incessant conflict for life and death; the stronger everywhere pursue the weaker, and thousands of animals daily become the prey of the wild and ferocious race, which precisely therein finds the means of existence; so that the death of the one is the condition of the self-preservation of the other: and yet amidst this endless annihilation the whole continues to exist.

How is it then, that, with this constant death and devastation, in the great economy of nature the equilibrium is so steadily preserved? Why do not the lions, the tigers, and the wolves exterminate the defenceless races from the surface of the earth; how is it that the eagles, the falcons, and other birds of prey do not extirpate the weak and tender-winged tribes; why do not the crocodiles depopulate the rivers, the sharks the seas? What, above all, are the means whereby the defenceless new-born progeny is protected from all these enemies, and in its helpless condition finds all its wants perpetually provided for?

To this apparent confusion, this omnipresent destruction, a limit is placed by the most adequate provisions. The ferocious animals are restrained in their wildness, where it is necessary, all their inclinations are regulated according to their wants, their passions are led and guided, and in default of knowledge, of foreseeing reason and a calculating understanding to discover for themselves the means for their own defence and of protection for their offspring, the Creator has bestowed on them the necessary gifts, has caused tendencies and instincts, adapted to the mode of life of each, to germinate from their organization, has thought for them, and has thus conducted the whole animal kingdom, as it were with invisible reins, with a wisdom and providence far above all human calculation.

The innate tendencies are emanations of a higher will implanted in the animal world, and are therefore perfect as the Source whence they have issued; they are the language in which the Creator speaks in nature, and therefore the true beauty of nature is not to be found in the brilliant colours of the flower nor in the splendour of the forest illumined by the rays of the rising sun, nor in the most beautiful landscape, but in the wisdom which radiates in all, in the thoughts of the Creator himself, which we may read in nature as in an open book, whence His wisdom and greatness are reflected as from a polished mirror, in a thousand glittering beams, but ever pure. The true and proper beauty of nature manifests itself most eloquently in the loving maternal care of the rising generation exhibited throughout the whole animal kingdom. A few examples will amply prove this proposition.

It is a generally acknowledged truth, that unity of object is always combined in nature with the greatest diversity and abundance of means of attaining that object. In no part of the whole creation is this more striking, than in the care taken for the continued existence of the various species of animals. The great difference, however, which prevails in the mode of life and necessities of the thousands of animals inhabiting this earth, must be attended with an equally great difference in the means whereby the object is attained.

Many animals live too short a time to be able to take care of their young, and are dead before their offspring have yet left the eggs; but neither are these forgotten—in their behalf, too, maternal love has been exercised.

Many, as the fishes, are too little attached to one place, in consequence of the necessity of seeking their own food, or of avoiding dangers, to be able themselves to bring up their young; but to the latter, also, nature is a careful mother. It is only among the higher animals, which from their organization and mode of life are better adapted to the purpose, that the education of the young is intrusted to the parents. By the former, the young are left to their fate; and here nature alone is, as it were, the mother: in the case of the latter, she resigns this care in part to the parents, by endowing them with maternal love and with the means of bringing up their offspring.

With a view to observe some order in the consideration of this rich field of nature, I shall, after a few general remarks on the care of the young which is manifested throughout the whole creation, mention some traits of the instinct and innate love of animals exhibited by the entire animal kingdom, from insects up to man, in the nurture of their young. Among the difficulties to be overcome by nature in bringing up the young, the provision, in sufficient quantity, of food adapted to their delicate constitution, deserves our first attention. This must not only vary for the several classes of animals, but must also differ from that which the stronger stomachs of the parents can digest. With this object nature has given to the mammalia the breast, where the young find in the mother's warm milk a food quite adapted to their still tender stomachs, and requiring but little effort to change it into chyle and blood. Some birds, particularly those which are graminivorous, she has provided with a crop, where the food is softened and prepared for the weaker digestive organs of the young; while the young of the lower classes of animals immediately after birth know how to select their food, which through the special management, or innate care, of the parents, whereof I shall hereafter speak, is always present for them in abundance.

A second very beautiful provision of nature is, that animals, at least in our regions of the globe, are born, not at all seasons of the year, but in the spring; hence they find the temperature and air their growth requires: they have no winter's cold nor keenness to endure, and are supplied with plenty of food adapted to their wants. In spring, the vegetable kingdom is sprouting up anew, the grass and leaves are still tender, soft, succulent, and abundant; the young of the herbivora therefore quickly find everywhere easily digestible nourishment, enabling them to thrive luxuriantly. Beasts of prey obtain a rich harvest in the abundance of other young animals, whose easily digestible flesh is quite adapted to the weak stomachs of their own offspring; the latter can exercise their not yet fully developed powers in catching the young of other species, while, if they had to contend alone with the parents, they must inevitably be overcome.

The same is true of insects and other lower animals, both those which live on plants and those which feed on other insects: in some the arrangement of nature for the preservation of the species is wonderful. The vine-fretter (*bladluis*), for example, which occurs so abundantly in our gardens, during the spring and entire summer brings forth living young, and all of the female sex; there is then abundance of food, and not an individual need perish for want: but in autumn, when the leaves fall off, and vegetation ceases, all would inevitably die of want of food, or be killed by the approaching cold of winter. A single night's frost would therefore, without some special provision, be sufficient to blot out this entire species from the list of living beings; but in the last generation their organization is so altered, that males too are brought forth, when, after pairing, no more living young ones are produced, but eggs, capable of enduring the severest cold, from which, in the spring, when the leaves, their food, have shot out, the whole race once more appears, which now, like their predecessors, are again viviparous.

A third important provision is the defence of the young against danger. Among the higher animals this is intrusted to the parents; but in many of the lower classes the young are, with some exceptions, neither taken care of nor defended by the parents, these being already dead. Other animals may consequently persecute the offspring with impunity, and the latter become helplessly a prey to the gluttony of the former; but, in addition to many other precautions, their destruction is prevented by the fact, that the more numerous the dangers are with which the animals or their young have to contend, and the greater the number of animals is which feed on them, the more their multiplication is increased; hence this is greatest among insects and some other of the lower classes of animals: thus one species of moth brings 200,000 moths yearly into the world. A nocturnal butterfly is said to be able to produce nearly a million and a half of eggs in the third generation. If the vine-fretter were for a year free from destruction, the earth would soon scarcely have room for all its descendants, as it is said to be capable of producing in the fifth generation about 5,000,000,000 of young, and at least twenty such generations take place in the year. This fertility is certainly astonishing, but by it the species is able to withstand the multiplied persecution to which it is exposed, and a profusion of food is provided for hundreds of animals, which live by their destruction.

In fishes, too, we meet with similar fecundity; thus the herring lays between 20,000 and 50,000 eggs; 383,000 have been found in a tench, 621,600 in a carp, about 1,000,000 in a thornback; in a codfish 3,444,000 eggs have been met with. As a great number of these eggs are devoured by other animals, and as many perhaps are not fructified, this loss is obviated by their great multiplication, and some provision is made against the destruction of the species. If the large herbivorous animals were as fruitful as the small, the vegetable kingdom would be devastated; did beasts of prey increase as much as the herbivora, or did large beasts of prey increase as rapidly as the small, excessive destruction would be the result.

Thus in nature everything is calculated beforehand, and the necessary equilibrium and order are maintained. Beasts of prey are the pruning-knife in the hand of nature, with which the superfluous sprigs are cut off, and growth and life are everywhere protected. Such animals, again, depend upon the abundance of their prey. Spitzbergen, for example, has scarcely a single insect on its plants, and therefore not a bird to feed on them. In Greenland we are already acquainted with more than twenty different insects, and here there are at least two species of birds which are nourished by them. In the warmest countries of America, where the number of insects is extraordinarily great, several hundreds of different kinds of birds are known, which limit the excessive augmentation of the insects, and live on them, to serve, in their turn, as a prey to other animals; thus all are harmoniously connected, and the fertility of one species is the source of life of another.

But should nature, in which so much plunder and destruction reign, be accused of cruelty, we find that even this is provided against. The poison of many animals appears rapidly to make their prey insensible, and to protect it from torture, and the tusks of the larger beasts of prey are so situated, and the animals themselves are so guided by instinct, that they always almost immediately bite through the great arteries leading to the brain, producing loss of consciousness in a few moments, and rendering death less painful. The much more distressing end from famine, want, and old age, is thus avoided, under which otherwise the animals would probably sink after a long period of suffering.

But it was not my object to indicate the mode in which the equilibrium of nature is maintained, but rather to adduce some proofs of the care taken by nature for the rising generation. Nothing affords such striking views of this, as the instincts and inclinations of the animals themselves, whereby they are so correctly and adequately guided in their actions, and in which we must above all admire the wisdom of the Creator, who has ruled all this with such perfection.

Where animals are still weak and tender, and too imperfectly developed to guide themselves, fixed innate tendencies are given them, to fill the place of reflection and of reason; where they are incapable of thinking for themselves, nature thinks for them. "Nature," says Herder, "thought for them, when she placed these inclinations in certain animals and not in others, and in its organization compelled the creature to see, desire, and do what she had pre-arranged for it."* If we now briefly trace from this point of view the animal kingdom from the insect up to man, we shall find maternal love of the offspring everywhere directed in the most admirable manner.

Among insects, for example, we meet with many species whose young cannot take care of themselves, and where the parents are dead before the future generation comes to life; without a special provision of nature, or arrangement on the part of the parents, these animals must perish; but in this case the mother provides for her offspring before it is yet in being, and seeks its food ere she has as yet laid an egg. Here too, as in higher classes of animals, the food which the young consume is almost always wholly different from that of the parents; but the female does not provide for her own taste or wants, but lays her eggs in the situations where her young ones can obtain the support adapted to them. The sand-wasp digs holes in sandy ground, in which she places a large spider or caterpillar, which she does not kill, but by wounding its nervous system in a particular point with her sting, only stuns and paralyzes, and thus prevents its corruption and putrefaction. Having done so, she then lays

* Herder, *Ideën zur Philos. der Geschichte*, 1 Th. p. 97.

an egg in each hole, where the young consequently finds its fresh food when it leaves the egg. But other wasps again from time to time open these passages, so made as to be undiscoverable to any others and carefully closed, so soon as the larvæ have consumed the provision laid up for them, which the parents seem to know exactly, lay fresh provisions in the nest, and close the latter accurately, to prevent the advent of any enemies. The wood-wasp brings to the ovum into the cell a sort of dough prepared by her, which is not adapted to her, but is the most suitable food for the larvæ emerging from the egg. The solitary wasp collects eleven or twelve little green caterpillars or maggots, although she herself lives on vegetable food: she attaches these to the egg, as if aware that the food she herself uses would be injurious to her young; the larva which comes out has in these just a sufficient stock of food suitable to the period which shall elapse until the perfect insect can support itself from the vegetable kingdom. According to Kirby and Spence, the number of the maggots depends upon their size: if they are large, the wasp uses only eight or nine; if they are smaller, eleven or twelve: but she always seeks full-grown maggots, apparently foreseeing that only animals which have attained their full growth could live long enough in the nest without food, while, if they were still young and growing, they would soon die of want and putrefy.*

Thus also the butterfly which produces the annular caterpillar, herself sucks honey from the flowers; but who has taught her, as she has never seen them, that her young, which are not to emerge from the eggs until the following year, will not require honey, but young leaves? She therefore lays her eggs not in the flower, but also not on the leaves, which fall off in autumn, but attaches them with a resinous matter, impermeable to water, around the young branches, as if she foresaw that the fostering vernal sun would make the tender leaves bud forth, so that the young caterpillar finds its food as soon as it leaves the egg.

Nor is the fact less remarkable, that the young ones do not leave the eggs until the fresh leaves have already begun to germinate, which takes place at very different times in different kinds of trees. Thus there is no apparent difference between the eggs of an aphis of the birch-tree and those of one of the ash; and still, under the same temperature and in the same climate, the eggs come out simultaneously with the budding of the leaves, a month earlier on the birch than on the ash, which so much later unfolds its youthful green. Spence took a branch from a birch-

* Kirby and Spence, *Entomologie*, Stuttgart 1823, 1 B. p. 382, *et seq.* (The English reader may find the original of the passage referred to in Kirby and Spence's *Introduction to Entomology*. London, 1816, 2nd edition, vol. i. p. 347.—TRANSLATOR.)

tree laden with such eggs, and placed it in water in his warm study, where the leaves expanded a month earlier than under ordinary circumstances, but with them the young aphides also appeared.* Who does not recognise in this wonderful harmony a true maternal care on the part of nature for the least of creatures, without which they would almost always, for want of the earliest and juicy green, miss their proper nourishment and perish? But those insects whose young ones come out in summer, lay their eggs on the leaves themselves, as if aware that in their case the precaution of attaching them to the branches was unnecessary.

Sometimes not only the food, but even the medium in which they live, is different. Thus the larvæ of dragon-flies, of the ephemera, of different kinds of midges, and of several other insects, live in water, which is absolutely fatal to the parents; the latter, however, overcome their natural dread of that fluid, and lay their eggs in it, frequently indeed risking their life to preserve that of their offspring. And where should I end, were I to attempt to describe the storehouses of bees, wasps, and ants, which are in themselves so worthy of consideration, but the description of which would exceed the limits of the present essay?

Some insects live to see the birth of their offspring, and the care of the latter is accordingly intrusted to the parents, by whom the young are tended with no less love than is manifested among the higher animals. Thus certain spiders enclose their eggs in a spun purse, which they fasten on their backs and carry about with them. If this purse be carefully taken off the mother, she leaves a long thread attached to it, and draws the eggs, so soon as they are let go, quickly to herself again, for the purpose of escaping with them. Bonnet placed one of these spiders before the conical hole of an ant-lion, a very voracious animal: the spider immediately attempted to escape, but the ant-lion seized the bag of eggs, which he endeavoured to bury under the sand. The spider turned to oppose him with all her might; at last the bag was torn off, whereupon the spider seized it with her jaws, and redoubled her efforts, but in vain; the ant-lion was the stronger, and buried both! The unfortunate mother could have saved her life by forsaking her eggs, but she preferred allowing herself to be buried alive, rather than that she should be separated from her offspring.† When the young spiders creep out of the bag, which is opened for them by the mother herself, they fix themselves upon the back of their parent, who carries them about with her for some time, and carefully tends them.

---

* Kirby and Spence, l. c., Band II. p. 485. (Vol. ii. p. 434 of the second English edition.)

† Kirby and Spence, l. c., Band I. p. 397. (Vol. i. p. 360 of the English edition already quoted.)

Even the scorpion, in other respects so generally abhorred, affords an example of maternal love: thus for instance, so soon as she apprehends danger, she opens her mouth wide, into which her very small and tender young ones creep; these she carries in her closed mouth into a place of safety, where, opening her jaws once more, she delivers herself of her beloved burden.

I might still adduce a number of striking illustrations of the point under consideration, but the foregoing may suffice to convince us, that even the most despised and neglected creatures, whom we often stamp with the name of vermin, proclaim to us the greatest wonders in nature, and prove that all creation is tended with equal love and goodness as well as with infinite wisdom and perfection.

Although fishes leave their eggs to their fate, they are nevertheless by no means destitute of maternal care. Impelled by their natural instinct, they seek by preference such localities to deposit them, as are exposed to the least danger, and afford, in the multitude of water-insects which throng these places, an abundance of food for their future young ones. Some, as the salmon, for this purpose swim up rivers, and with amazing force and agility succeed even in leaping up waterfalls, of which I was myself once an eye-witness in the case of a trout. The salmon then dig a pit in the sand, in which they lay their eggs, and cover them up, whereby they are kept from being washed away and separated from one another.

In the river St. Lawrence above the waterfalls of Niagara, many fishes even make little dams on the strand, of small stones, which they collect with their mouths, to prevent the eggs, laid behind them, being washed away by the force of the current.

Some species of stickleback (*Gasterosteus*) go so far as to construct nests, just like birds, in which the eggs are deposited. The male builds an arched nest of vegetable fibres and filamentous seaweed, and glues it with the slime of his body, first fastening the foundation by constantly pressing it in more firmly by rubbing it: if a branch or stalk in the structure does not answer, he draws it out and substitutes another, then covers the nest with an arch, and bores a second opening in it, so that he can swim through. When this is accomplished, he seeks among the neighbouring fishes of his species a bride, just then adorned by nature in unusual colours, and conducts her to his nest as to a bridal chamber, where the eggs are then laid. When this is done, he fetches a second wife, and so, according to the statement of Coste, these nests sometimes contain from 1000 to 2000 eggs.*

But in contrast to what we observe in other animals, the male

* M. Coste, *Instructions pratiques pour la Pisciculture*, 1853, pp. 67 and 74.

alone keeps guard over the nest, in which the female has no exclusive share; on the contrary, the females are even his most dangerous enemies in endeavouring to devour the eggs. It is then his hard task indefatigably to defend the latter for a month against the frequently repeated attacks of these plunderers, during which time he does not leave the nest, but incessantly cares for all. He begins by first strengthening the nest, stops one opening closely, and covers the nest with little stones, often half as large as his own body, and which he fetches with difficulty from the neighbourhood. He has then only one opening to defend, and keeps continually before it, making a constant movement with his anterior fins, so as to keep up a perpetual stream of fresh water over the eggs, which would otherwise spoil and not come to maturity. During this time he drives away every other fish or female which comes too near his nest. If the number of his enemies is too great, he employs cunning, and pretends to dart upon some prey, withdrawing for some moments, whereupon his enemies, eager to participate in the unseen booty, follow him; he thus entices them from his nest, but quickly returns to his beloved habitation. If he succeeds through these unwearied efforts in preserving his treasure until the young are on the point of coming out, he redoubles his exertions; he again removes the little stones, makes several openings in the nest, increases the constant stream of water, and places the eggs at one time near the side, at another near the middle of the nest. If, after ten or twelve days, the young fish have come out, he is obliged still for a long time to protect them from enemies: at first, on account of the heavy yolk-bag adhering to them, they can with difficulty move; but he does not suffer one to leave the limits of the nest, or, if that happens, he brings it back in his mouth; if many deserters follow, he sometimes seizes a number of them together with his mouth, but without injuring them. As his numerous family become larger, they require more room, and he now allows them to swim in the immediate vicinity of the nest, but, like a shepherd's dog, swims constantly about them, to keep his flock together, until, after twenty days, he can leave them to themselves: although under other circumstances voracious, he has during all this time allowed himself no food. Can the maternal care of nature express itself more strongly than in this insignificant fish?

Nor is this maternal care of nature deficient in the amphibia, although they must almost always leave their young ones to their fate. In crocodiles, this care is carried further than in many others. According to van Humboldt* they seek their eggs,

* *Reise in die Aequinoctial Gegenden:* Stuttgart, 1815: tome iii. 427; also Burdach, *Physiol.*, 3 B. p. 117.

covered up in the sand, towards the time when the young come out, call their young, which answer them, lead them to the river, and protect them against danger. But this the tortoise, which has in its shield the means for its own defence, but has received no weapons from nature, cannot do. But nature is not therefore exhausted of her means for the defence of the offspring. The sea-tortoise, for example, likewise digs a hole in the sand on the shore, and covers this again to let the eggs be hatched by the rays of the sun ; but scarcely have the young ones left the eggs, when a variety of birds and other animals pursue them. But who must not admire the instinct which impels them immediately to betake themselves to the sea, and so to elude their enemies? If they be stopped in their course, and an attempt be made to divert them and make them take another direction, they immediately turn back to repair to the sea, which they have never beheld. Who has taught them the way and given them a compass? and who does not admire the incomprehensible instinct of nature by which they are involuntarily impelled to watch for their self-preservation?

But nowhere do we find the most tender tokens of love and care, a constant eagerness and attention for the offspring, for which they seem, as it were, alone to live, more evidently and strikingly marked than in birds.

Here we already observe a higher development of parental love ; their care is greater, their passions are more noble, and are still further elevated by a remarkable peculiarity, which we meet with less frequently among the lower animals, namely, a conjugal connexion—a sort of marriage. Who does not recognise in this respect our doves, and especially the turtle-dove, so often sung by poets, as examples of mutual faith and love? And in this the special care of nature again appears, that we may assume it as a general rule that the impulse to this mutual union is peculiar to those animals whose young ones need, in the earliest period of their life, the care of both parents ; in birds, as ducks and hens, whose young ones immediately find abundance of food, this was not so necessary ; but where the food must be brought from a distance, while the unfledged young ones cannot long be deprived of the warmth of the mother, the care of both parents was indispensable, and here we find this connexion, or rather marriage, instituted by nature herself.

This matrimonial connexion lasts in some animals as long as the common care of the young requires it, and does not cease until the latter are full grown ; this is, for example, the case with some mammalia, as the bat, rats, and rabbits. Thus also with many birds of prey and singing birds, as well as with the raven ; at the time of emigration the pairs separate, but appear to unite again in the following year; in others, as the eagles and doves, and among the mammalia, foxes and deer, this connexion lasts through the whole of life. Of this a striking example was afforded by a stork whose wife had been prevented by a wound from undertaking the journey; during three consecutive years he sought her each spring, and then remained with her the following winters also.* This connexion is very close in parrots, and particularly among the so-called *inseparabiles*. Bonnet had kept a couple of these birds for four years, when the female fell from old age into a state of debility, and could no longer reach the feeding trough, but was now fed by the male; and when she became unable to get on the roosting perch, the male endeavoured with all his might to help her up. When she died, he ran about her in great distress, endeavoured to give her food, uttered a mournful cry, and died himself a short time after.† Nature even decorates many birds at pairing time with feathers of unusual splendour, as if she wished thus to heighten conjugal love. Others sing their good fortune in most melodious tones; thus the turtle-dove coos, the lark rises singing in the air, and the nightingale utters his beautiful songs, while his spouse is brooding. But as soon as the young are brought forth he is silent, as if he feared to betray the proximity of the nest, and now he helps to feed his offspring. In this the parents even use the greatest caution, and never fly directly to the nest, but conceal themselves at some distance in the bushes, so as to steal unobserved to their young, whom they leave again with equal circumspection.

Many birds even remove their young when they observe that they are discovered; thus, if one of the young be taken out of the nest of a night-owl, the parent will carry away the remaining little ones in the course of the following night; the same we often see in dogs and cats. The cunning with which birds endeavour to decoy enemies from the nest is equally remarkable; for example, if a dog or a man approaches the nest of a partridge, the male first flies up with a cry of terror, and thus warns the female, but quickly falls to the ground with dependent wing, as if he could not fly or was wounded, and so allures the enemy by the hope of an easily gained booty from the nest, while the female uses the moment to escape with the young. The same is met with in other birds. Coste relates that he was himself once the dupe of the cunning of a lark, which he saw suddenly roll before his feet and with difficulty steal away, as if it had no strength to move; he bent to take the animal in his hand, but at the moment when he thought to seize it, it made a fresh effort, though it ap-

* W. Vrolik, *Het leven en maaksel der dieren*, 1 d. p. 58.

† Burdach, *Physiol.*, 1 B., p. 366.

peared to be able only with great difficulty to remove a little farther. Thus the bird enticed him to an adjoining field, and when he thought he had brought his pursuer far enough from the track of his nest, flew at once joyfully and briskly up, and made his triumph at the result of his cunning known in lively songs.*

Bonnet mentions swallows which flew even into burning houses to save their young or perish with them.†

But the maternal care of nature is visible in birds chiefly in the construction of their nests, which they prepare with an industry and art sufficient to surprise any one who reflects upon the instruments they have to work with. In this case the bird provides for its young before a single egg is laid.

In most birds the male brings the material, and the female builds the nest. Birds' nests are, like all artificial products which are prepared by instinct, perfect of their kind, and deserve the name of masterpieces. Where nature is the teacher and the assistant of the undeveloped intellect of animals, everything is equally beautiful and adequate, worthy of her, perfect, and above criticism. Some general observations and examples selected from the abundance of matter at our hands may suffice to establish this proposition.

Birds' nests are always calculated according to the number and size of the young ones, and in this no bird is liable to err ; small eggs, which cool more rapidly, require a more persistent warmth, and therefore the smaller birds build deeper nests, and the eggs lie on a softer and better heated bed, in consequence of which they do not cool so quickly when the birds fly away. Thus the nest of a lark is much deeper, and the eggs are warmed to a higher degree than that of a stork or goose. They line the inside of the nests with substances which are bad conductors of heat, such as straw, moss, hair, down, or feathers. The care of the crossbill (*Loxia curvirostra*) of northern countries is remarkable in this respect. This bird lays her eggs in January, when rain and snow cover the earth, because the fir seeds, the food of her young, exist in abundance then, and not in spring. But her nest would be soaked with the constant wet, and hatching would be rendered impossible by the cold so produced, were it not that nature has taught her to smear her nest with resin, as if she knew that this makes it impervious to snow and water.

But provision is made in the most ingenious manner by birds in the construction of their nests not only for warmth, but especially for the protection of the eggs and young ones. The nests are, in fact, more complicated and better adapted for defence in proportion to the danger to be apprehended, and their architec-

* Coste, l. c. p. 78.

† Burdach, *Physiol.*, 3 B., p. 124.

ture is even completely modified according to the particular enemies the occupants have to fear, Thus our singing birds usually build their nests in the thickest foliage, or in the hollow of a tree, where they are scarcely visible or accessible to birds of prey. This would, however, not secure birds in the warmer regions of the globe from the attacks of apes and serpents, which in great numbers persecute them; but many of these birds build their nests in the extreme branches of trees, frequently in such as overhang water, which are beyond the reach of their enemies.

The Bengal crossbill, not content with this, prepares a cord nearly a yard in length, constructed of vegetable fibres and dry stalks of grass, which he attaches to the extreme branches of trees above the water, and on this he suspends his nest, which is then swung to and fro by the wind, and is unapproachable by any other animal.

In some of these nests the opening is at the sides, in others it is even turned downwards towards the water, over which it hangs, and leads by a side passage to the young. The tailor bird fastens three leaves of a tree with cotton thread to one another, by means of its beak, and makes a knot at the end of the thread to prevent it slipping off; this nest is scarcely distinguishable from the other leaves of the tree.

In Abyssinia it often rains incessantly for many months, with west winds, but the *Loxia Abyssinica* builds its nest so that the opening is always turned to the east, and the eggs are protected above from the rain by an impervious covering. Our swallow attains the same object, by suspending his artfully constructed nest on the beams of our houses. In the East Indies a species of swallow forms the well-known edible nest with the mucus of its stomach, and attaches it to almost inaccessible rocks.

The mode of life and feeding are also kept in view, and many birds accordingly build their nests where they can most easily get their food; thus the eagle and other birds of prey construct them on lofty rocks and trees, whence they have an extensive view, and can easily perceive from a distance the little game; the lapwing and others build on the soft ground, where they can readily find the worms on which they live, and select in preference the side of a ditch or channel, where they can leave the nest unobserved. Water-birds build their nests on the shore, or even construct floating nests on the water, so that there can be no danger of drowning in case of excessive floods.

Proportionate to this is the care with which birds cherish their eggs or offspring: they do not mind their want of weapons, but often defend them at the risk of their lives. The little humming-bird, which is occasionally a prey to a certain species of spider, defends its eggs with such fury that it flies in the face of any one who approaches them. Among many birds of prey the defence of the young is intrusted to the mothers, but in such cases the females are larger and stronger than the males. They hatch the more constantly the nearer the young are to coming out, as if they knew that cooling is then much more dangerous to the latter; accordingly wild ducks, which at other times are so timid, then often let themselves be taken on the nest in the hand. Some birds, especially those whose eggs are white, as ducks, cover their nests, when they leave them, with hay or leaves, to prevent them from cooling, and to conceal them from enemies; the lapwing does not do this, its dark and green eggs are nearly the colour of the grass and earth, and therefore do not strike the eye.

The maternal care of nature is also particularly visible in the different development of the young on leaving the shell. Thus the young of those birds which build their nests on the ground, as hens, ducks, lapwings, and others, come into the world with tolerably strongly developed feet, and very quickly leave the nest; they then either repair forthwith, like young ducks, to the water, and seek food for themselves, or follow the mother, who lays their food before them, and with peculiar tones invites them to use it, or warns them of approaching danger. But if the young of those birds which build their nests in trees or on lofty rocks were to leave the nest immediately, they would fall down and be dashed to pieces; this is, however, also guarded against, inasmuch as the latter come into the world in a much more imperfect state, as all the eggs of these birds are considerably smaller, and the young are therefore less developed when leaving the shell, being for the most part naked and blind, and their feet being so weak that they cannot run until they are able to fly. This singing birds and birds of prey are seldom able to do until after the lapse of two or three weeks. The young of fowl and waterfowl are very soon able to find their food, and to avoid danger, but do not fly for two or three months; in the first class mentioned the wings are more developed than the feet, in the second class the case is reversed; in the latter (hens, &c.), the care of both parents is not so necessary, and the duty is therefore for the most part left to the females, and in many instances the male even leaves the female at the time of hatching, to return towards autumn.

In singing birds and birds of prey, the male and female hatch and take care of their tender young ones alternately, particularly when the food is difficult to obtain, and, as often occurs, is only to be had at a great distance. Sometimes even, as has been observed in woodpeckers and owls, the male has taken on himself exclusively the care of bringing up the young, when the female was captured; so that this beautiful natural instinct is adapted even to meet accidental and unusual circumstances.

Among some birds, as divers and waterfowl, the male assists in hatching, but does not trouble himself about the young, which, under the guidance of the mother, find everywhere a sufficiency of food; on the contrary, in most coupled singing birds and birds of prey, and consequently in the case of the heron, the male takes no part in the hatching, but feeds the young, as the mother would be obliged to leave the nest too long if she went in search of food. In singing birds the parents remain long in the neighbourhood of the nest, even when the young no longer need their care. A wag-tail which had hatched a young cuckoo in a hollow oak, through the narrow opening in which the nursling could not escape, even stayed behind from her autumnal migration, and fed the cuckoo in the winter. Thus instinct is adequately modified according to circumstances, and is quite adapted to the wants and mode of life of the animals.

The first act of the mother after the young ones have come out, is to free the nest from the egg-shells, which might easily injure them, nor does she seek their food until this has been accomplished. The birds which live on insects break these up and feed their young with them; birds of prey just soften the meat in their crop, to make it more easily digestible; at a later period they lay dead animals before their young, afterwards such as are more or less wounded, and lastly small living animals, so that they may exercise their powers in catching them. Birds living on seeds, as doves, first soak them in their crop, and give them to their young into their beak; sparrows and others feed their young at first with insects, as if they knew that the grain on which they themselves live was as yet indigestible for their weak stomachs. Thus a pair of sparrows for example, according to Bradley's observations, devoured, at the time they were feeding their young, 3360 caterpillars in a week. They observe strict order in feeding, so that none is forgotten or doubly fed at the expense of another, but each gets his proper share in turn. But as if all these precautions were not sufficient for the preservation of the species, nature comes to the rescue when the young ones are destroyed by birds of prey, and confers on the parents the curious property that, under these circumstances, they can again lay eggs, which otherwise they do not. They then build with unwearied industry another nest, hatch once more, and so repair the loss they had sustained.

And thus we approach the mammalia, at whose head man, the end of the visible creation, is placed; and here, too, we observe no less striking evidences of maternal care. Conjugal union is here, however, less general than among birds. The young mammalia do not all need the care of both parents, as the mother's milk is sufficient for their earliest wants; the herbivorous mammalia find abundance of food in spring, and the young are soon in a state to provide for their own necessities, and on the approach of danger to take refuge under their mother's protection. But among the carnivora, the young are by blindness and weakness of the limbs for a time confined to the nest, just as we have seen in birds; and how could the mother procure a steady supply of food for herself and her young ones but by constantly leaving the nest and exposing her young to danger. Here, therefore, the male again assists; he exchanges his tiger-like rage for love to his spouse and offspring; one now defends the young while the other goes marauding, and constantly brings in fresh supplies of food.

They, too, make their nest or den, as we remarked of birds, in the most secret places. The lion renders the path to his young imperceptible by frequently running to and fro, or sweeps out its traces with his tail. The male fox constantly drags food for his wife and young ones to his den, but lets no bones lie about, nor does he even rob in the neighbourhood of his abode. The pole-cat leaves his excreta at a distance, and also carries off those of the young, that the nest may not be betrayed by the smell.

Beavers make their ingenious structures, in which they keep their stock of the necessaries of life, before they bring their young into the world. They first replenish their magazines, and both subsist on the stores they have laid up, but so soon as the young are born, the male leaves the supply to his wife, and seeks his own food elsewhere; he does not, however, entirely separate from the female, but visits her frequently.

The special care and arrangement of nature are particularly and strikingly exhibited in the marsupial animals, whose young come into the world prematurely and in a very imperfect state, thus enabling the animals to pair twice in the year; but in this case the young ones are kept in a pouch, as in a nest attached to the mother's body, where in her milk they find food, while the pouch affords the requisite warmth and protection. If the young are sufficiently grown to leave the pouch, they jump in case of danger on the mother's back, and throw their tails round her tail, when she escapes with her beloved burden.

As we ascend in the scale of the creation, we find the passions and inclinations of the animals attain a higher degree of perfection, consequently the traces of reciprocity of love to the parents now begin to be more distinctly manifested. A young walrus does not leave its mother even when she is dead; in a lamb I once observed the most evident disquietude on the death of its mother, whose body it incessantly pawed, as if it wished to rouse and call her back to life, while it clearly exhibited its embarrassment, pain, and uneasiness in its bleating and movements.

Thus a story is told of an old, blind, decrepit rat, which was drawn along by its young ones towards some crumbs of bread, and on the approach of noise was again brought back by them into safety.

The duration of this filial love varies. Mother and young forsake one another when the young can fly, or when the mammal ceases to suck. Among divers the young remain with the mother until the autumnal migration, and only the males travel alone. Among herbivorous animals, where there is abundance of food, this connexion lasts longer than with the carnivora; the large birds of prey drive off their young at an early period to hunt for themselves. With birds or beasts of prey living in the wild state, the connexion ceases in the autumn or winter, when food becomes more scarce. Thus nature arranges and disposes everywhere, and demonstrates her maternal care in unmistakeable language.

My limits forbid me to quote a number of others out of the many examples which occur to me of the care and love of animals for their young; I shall, however, adduce one or two instances to show that these principles exist in several animals in a degree of sensitiveness sufficiently striking to put many a human being to shame.

A cat belonging to a friend of mine had placed her kittens in a cradle; upon which the young ones were taken from her, and the cradle was put out in a pond to be cleaned; shortly afterwards the cat disappeared, and when, after some time, the cradle was again taken out of the water, she was found in it drowned. She had overcome her natural aversion to the water, and had swum to the cradle, where, expecting to save her young ones, she had lost her life. Should we not think such an instance of maternal love in a human being worthy of a statue?

The maternal love of apes is well known. A striking incident related by the distinguished traveller Poeppig, in the account of his voyage to Chili and Peru, may here be introduced in his own words. He says: "What has been told of the extraordinary maternal love of apes is really true, and I have myself been a witness of a proof of it, which for a long time deprived me of any wish to hunt them. In order to obtain a young Coaita ape, which I wished to rear, I had, among one of the densest troups slowly advancing through the tops of the trees, selected for my aim a female, which carried a tolerably large young one pressed to her bosom. It was long impossible to get near enough to the cunning animal, from which all the others, as if they were aware of the danger, fled away. The first shot wounded her in the hind feet, and compelled her to move more slowly. The second struck more important parts, though without killing her; but it distressed me much to observe, when the animal, as the smoke slowly passed off, became visible on a slight branch, that at the moment she was aimed at, perceiving the danger to her young, she had rolled herself together over it, and had thus received the entire discharge. Soon the last agony began, but in place of suspending herself, after the manner of the males, by the tail, and so exposing her young to the danger of a violent fall, the dying mother let herself glide down along a creeping plant to a thicker branch, where she cautiously laid down her burden, and immediately after fell dead at my feet. Never since that time have I shot at female apes."*

If we now cast a glance back at what has been stated, who does not recognise in nature the image of a careful mother, who loves all her children with an equal love. In most insects the propagation of their species is the latest object of life, and dying they resign their offspring to nature. Not one of them is forgotten, they are all maternally cared for; the tender caterpillar at its birth finds the young leaf, its food, provided for it, just as the newly-born infant meets with the well supplied breast of its mother. The higher animals indeed take care of their offspring, but in this they are directed by instinct alone; their fury is changed into love, and their timidity into courage, and they become as children in nature's leading-strings. But only man, placed as he is at the head of the scale, can dispense with this guide; he alone can watch for his children and himself, govern and bring them up to higher moral training.

And should, then, man alone, as he comes into the world a weak and helpless child, ignorant of everything, without any other instincts than such as are required for his animal existence, though with a higher destiny, be treated by nature with the cruelty of a stepmother?

No; here too, where all is arranged according to a quite different and higher plan, the beneficent and loving, but more elevated objects of the Creator are drawn for us in well-marked lineaments.

Indeed, I repeat, among the lower animals, nature takes all the care upon herself; here she alone is the true-hearted mother, who provides for all; among the higher animals she resigns to the parents the care of feeding and rearing the young, but retains to herself the direction of the passions and inclinations, and the development of the mental powers and thoughts, which the Creator himself has implanted in every animal, modified according to its wants. In man alone the Creator has left the development and education of both body and soul exclusively to the parents. He has given to the child only capacity and disposition, but to the parents their acquired knowledge and under-

* Poeppig, *Reise in Chili, Peru, und auf dem Amazonenströme,* Band II. p. 236.

standing, excited by parental love, and ennobled by innate moral sentiment, and the consciousness of a higher origin.

Not the animal but man must mould himself, and raise himself to a higher degree of humanity and of moral worth, must perfect himself for virtue and piety, yea, even for immortality. His intellectual powers must be fettered with no bonds of instinct, and therefore is he created without innate knowledge, but at the same time free to develope himself into an independent thinking being, and by his own power and exercise, by exertion and contest between right and wrong, to prepare and form himself for a higher state. For this purpose nature has prolonged as much as possible his youth and pupillary age, to an extent which occurs in no one of the inferior animals, seeing that he has everything to learn, even to his own language, which is given innately to every other animal according to his need. Nor must he be the born servant or obedient slave in nature's leading-strings, but a beloved and free son in the house of his Father, whose image he, and he alone, bears in his bosom. To such great objects, then, is maternal love in man proportioned; she is nobler, higher, her aim is loftier; she contemplates not merely bodily education, but the moral and intellectual development of the spirit; she scatters in her beloved children the seeds of knowledge, of virtue, of piety and love, she leads humanity to the perception of the true, the beautiful, and the good; in a word, her looks extend beyond the grave.

And who that understands and feels this elevated language of the Creator in nature, proclaimed as it is to us with loud voice, can still inquire whether in nature lessons of pure and lofty morality and genuine humanity are preached? How can any investigating and reflecting mind doubt that there is a bible in nature, which is surely written by the Creator himself, and the fundamental text of which is still kept by him pure and inviolate, and in the state in which it issued from his hand?

And should, then, these last and elevated sentences in the book of nature alone prove to be an adulterated text, must we in the innate feeling of a higher moral tendency in man, which can here never attain to perfect maturity, recognise a lie? Has the Creator then implanted in us a deceitful phantom, which points us to higher regions of moral perfection which do not exist? Or has He here placed the cup of immortality and perfection to our mouth, in order, when we have tasted it with our lips, to draw it for ever back from us, when we wish to enjoy it? No! where nature speaks thus, there is no room for falsehood.

The proposition of the excellent Herder is very just, "whether the Creator erred in the object He proposed to us, and in the organization, which for the attainment of that object He so ingeniously constructed, or whether that object extends beyond our present existence, and this earth is only a place of transit, a school of preparation."* Well does Tollens impressively ask :—

Say, when God came down,
And asked the prostrate earth,
Where her Creator's image,
Most faithfully was shown;
Say what could then be chosen,
Or offered to her God,
Except a tender mother,
Her infant at her breast?

But strikingly as this is expressed, I do not think that which she possesses in common with the beasts can be the true, or most elevated characteristic of a mother.

No; too often have I been the unobserved and silent witness of a nobler spectacle, as I have occasionally with rapture watched an excellent mother, surrounded on all sides by her offspring, who with open ear and eye hung upon her words, sowing the seeds of knowledge, virtue, and piety, and endeavouring to develope more fully the moral feeling, and so to bring her children nearer to Him, from whom she had received all.

And should this be a false, a supposititious case in the book of nature?

Or, where the chisel on a mother's grave has carved the touching words of faith: "Father, here am I, with those whom Thou hast given me, to Thee I thankfully restore them." Could this inscription be a fiction, and heaven so mock the exalted maternal love implanted by itself?

No, mothers! then here be conscious of your high position and lofty calling upon earth. The Creator has taken upon himself the rearing of the animal kingdom, and has guided them with loving care, by endowing them with innate tendencies and instincts, adapted to all their earthly wants. But the reins for the moral formation of man He has placed in the gentle, loving, mother's hand, that under her care and guidance the nobler blossoms of heaven might bud and flourish. Mothers! here you occupy the place of the Most High, for the higher moral development and civilization of the human race He has resigned to the faithful mother's heart, which He had formed for love and piety. Here is then your noblest task and sphere of action, but here you are the genii of mankind, messengers and angels of the Highest, who seeks to guide us to genuine humanity and higher cultivation, to excite in us the sentiment of the true, the beautiful, and the good, to form us for virtue, piety, and love, and so to

* *Ideen zur Philos. der Gesch.*, 1 B. p. 183.

lead us to higher realms, whence every good and perfect gift has descended to man.

If it is then the language of the Creator which we read in the book of nature, where everything bears the mark of the most elevated love and truth, perfection and order, let us, who can penetrate only to the outer covering of nature, gaze with reverential admiration at the wisdom, love, and greatness of the omnipotent Maker, who has created all with the word of His power—"Let these things be."

---

